# Can workplaces be predictors for recent onset latent tuberculosis in health care workers?

**DOI:** 10.1186/1745-6673-4-20

**Published:** 2009-07-24

**Authors:** Kittisak Sawanyawisuth, Naesinee Chaiear, Kanlayanee Sawanyawisuth, Panita Limpawattana, Janpen Bourpoern, Wipa Reechaipichitkul, Ken Takahashi

**Affiliations:** 1 Department of Medicine, Faculty of Medicine, Khon Kaen University, Khon Kaen, 40002, Thailand; 2 Department of Community Medicine, Faculty of Medicine, Khon Kaen University, Khon Kaen, 40002, Thailand; 3 Department of Biochemistry, Faculty of Medicine, Khon Kaen University, Khon Kaen, 40002, Thailand; 4 Infectious Control Unit, Srinagarind hospital, Faculty of Medicine, Khon Kaen University, Khon Kaen, 40002, Thailand; 5 Department of Environmental Epidemiology, Institute of Industrial Ecological Sciences, University of Occupational and Environmental Health, Japan

## Abstract

**Objective:**

To study the association of workplaces and recent onset latent tuberculosis (LTB) in health care workers (HCW).

**Methods:**

A case-control study was conducted at Srinagarind Hospital, Khon Kaen University, Thailand. We recruited HCW who had results of tuberculin test within 2 consecutive years from 2001–2008 and also had fixed workplaces (working hours more than 40 hours/week). Cases were subjects with tuberculin conversion, while controls were subjects with negative results of tuberculin test in two consecutive years. Tuberculin conversion was defined if a subject had a negative baseline tuberculin test and a positive tuberculin test in the next consecutive years. Baseline characteristics, workplaces (office, in-patient unit, out-patient unit, intensive care, operating room, and laboratory unit), tuberculosis related variables, and prevention strategies were studied. Multiple logistic regression analysis was used to identify predictors for tuberculin conversion.

**Results:**

There were 624 subjects who met the criteria and 163 subjects had tuberculin conversion (26.1%; case group). The median age and male/female ratio of both groups were 39 years old and about 1:4. The cases group had higher percentage of subjects who worked at in- and out-patient department (30.7 vs 20.2 and 17.2 vs 12.2, respectively), had history of tuberculosis exposure in the past year (32.1 vs 16.1), and had history of prevention by any method and by surgical mask (49.4 vs 37.0 and 54.3 vs 38.3, respectively). Workings at in- and out-patient unit and history of tuberculosis exposure in the past year were significant predictors for tuberculin conversion (adjusted odds ratio and [95% confidence interval] of 1.99 [1.25–3.17], 1.91 [1.10–3.17], and 2.26 [1.47–4.96], respectively).

**Summary:**

Workplaces in health care facilities do increase risks of LTB in HCW, particularly in in- and out-patient unit. Policy development regarding tuberculosis infection control programs focused on workplace prevention in health care facilities in Thailand is needed.

## 

Latent tuberculosis (LTB) is the stage of *Mycobacterium tuberculosis *that is asymptomatic, dormant and non-contagious [1]. A positive for the tuberculin skin test (TST) is the evidence of LTB as a prevalence case. The TST conversion from negative to positive from one year to the next indicates recent onset LTB or an incidence case. This individual is at risk to develop active tuberculosis in the future [2,3]

Health care workers (HCW) are considered as high risk for LTB [4]. The prevalence of LTB in HCW is different from country to country [5-7]. In addition, its prevalence may vary by places of work. HCW who work in a bronchoscopy room[8] or serve particular patients such as tuberculosis or HIV patients might have a higher risk for LTB. Tuberculosis infection control programs should be emphasized in health care facilities [9]. There are limited numbers of studies in this country assessing the incidence and predictors for recent onset LTB in specific health care facilities. We evaluated the association of various workplaces in health care facilities and recent onset LTB by a case-control study in an endemic area of tuberculosis.

## Methods

### Study population

The study was conducted at Srinagarind Hospital, Khon Kaen University, Thailand. We recruited HCW who had results of TSTs from 2 consecutive years from 2001–2008. Cases were defined as subjects with TST conversion, while controls were subjects without TST conversion. TST was done by using the two-step technique.

We enrolled HCW who worked continuously at least 8 hours in 5 different locations in our hospital including, hospital office, inpatient unit, outpatient unit, critical care unit and operating room. Exclusion criteria that was applied tosubjects of both groups included history of recent or active tuberculosis, suspicion of tuberculosis by previous chest X ray, diabetes mellitus, HIV infection or having received immunosuppressive therapy or steroids. These factors may contribute to a false negative TST due to the suppression of immune system, while people with tuberculosis infection will already have a positive TST.

The TST was given by injection of 0.1 ml of 5 tuberculin units of liquid tuberculin intradermally on the forearm. A subject's forearm was examined independently by two infectious disease control nurses 48–72 hours after the injection. The reaction was seen as an area of induration around the site of the injection. The diameter of the indurated area was measured in millimeters. The average diameter of the two readers was reported. An induration of 10 or more millimeters was considered a positive reaction. If the TST was negative, the TST was repeated within the next three weeks to eliminate the boosting effect. This phenomenon occurs in people who are skin tested many years after becoming infected with M. tuberculosis. An initial TST may be negative, followed by a positive reaction to a TST given up to a year later; this happens because the first TST boosts the immune response.

TST conversion was defined if a subject had a negative baseline TST and a positive TST in the next year. In addition, the TST conversion was also defined if the induration of the second-year TST was more than 10 millimeters and greater than the first-year result that was initially greater than the 10 millimeters. The TST was repeated in 12 months later for those with a negative first-year TST. An annual TST surveillance of health care workers is not a standard practice in Srinagarind Hospital nor most health care facilities in Thailand.

### Data collection

We recorded each subject's data on an infectious control unit chart including the baseline characteristics such as age, gender, and duration of employment, working unit, duration of employment in years, presence of bacillus Calmette-Guerin (BCG) scar, previous history of tuberculosis in family members or colleagues, previous history of tuberculosis exposure at workplace in the past year, previous history of previous TST, and previous history of using surgical, N95 or hepa masks. The frequency of mask use was defined as either, using at all times or occasionally while working.

### Data analysis

Baseline and clinical characteristics of cases and controls were compared using descriptive statistics. Wilcoxon rank-sum or Students t-test and Fisher's exact tests or Chi-square test were applied to compare the differences in numbers and proportions between the two groups.

Univariate logistic regression analyses were applied to calculate the crude odds ratios of individual variables for the development of TST conversion. All variables with p values < 0.25 in univariate analysis were included in subsequent multivariate logistic regression analyses. All variables with p < 0.10 were retained in the final model by the backward elimination technique. Analytical results were presented as crude odds ratios (OR), adjusted OR, and 95% confidence intervals (CI).

The goodness-of-fit of the final model was evaluated using Hosmer-Lemeshow statistics [10]. To evaluate the discriminatory power or accuracy of the model, *c *statistics or area under the receiver operating characteristic curves were examined [11]. All data analyses were performed with SAS software version 8.2.

## Results

**Figure 1:**
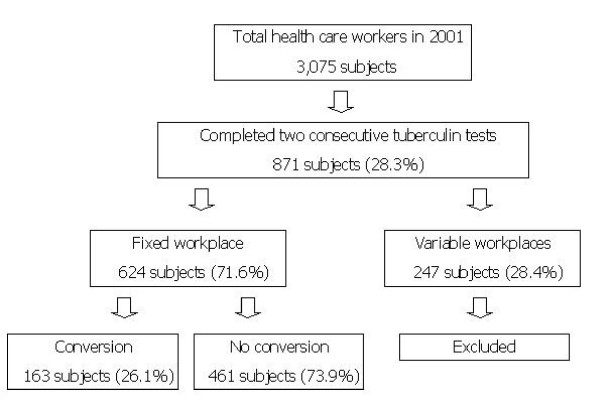
**Flow chart showed enrollment process**.

In 2001, there were 3,075 health care workers in Srinagarind Hospital and 35 subjects were excluded due to history of recent or active tuberculosis. Of the remainder, 871 subjects or 28.3% had TSTs in two consecutive years. In total, 624 subjects or 71.6%, were eligible, while 247 subjects, 28.4%, were excluded due to variable or rotating workplaces (Figure 1). There were 163 subjects, 26.1%, who had TST conversion and became the cases group.

The baseline characteristics and variables related to tuberculosis of the case and control groups are presented in Table 1. The median age of both groups was 39 years-old. Male subjects accounted for about one-fourth of the cases group. The case and control groups were significantly different in working units, history of tuberculosis exposure in the past year, and history of prevention by any method or by surgical masks.

**Table 1:** Baseline characteristics of subjects with and without tuberculin test (TST) conversion

Variables	No conversion N = 461	Conversion N = 163	p value
**Baseline characteristics**			
Median age (range), years	39 (19–58)	39 (17–59)	0.9972
Male gender	95 (20.6)	40 (24.5)	0.2946
Median duration of employment, years	14 (2–31)	13 (2–27)	0.9302
**Working unit**			0.007
Office	235 (51.0)	56 (34.4)	
Inpatient unit	93 (20.2)	50 (30.7)	
Outpatient unit	56 (12.2)	28 (17.2)	
Critical care unit	20 (4.3)	7 (4.3)	
Operating room	39 (8.5)	13 (8.0)	
Laboratory unit	18 (3.9)	9 (5.5)	
**Tuberculosis related variables**			
BCG scar	284 (61.1)	102 (62.6)	0.8263
History of tuberculosis in family	27 (5.9)	19 (9.9)	0.0825
Years of tuberculosis in family, years	8.5 (0.5–36)	8 (0.5–30)	0.8392
History of tuberculosis in colleagues	32 (6.9)	9 (5.6)	0.5406
History tuberculosis exposure in the past year	74 (16.1)	52 (32.1)	<0.001
History of previous TST	162 (35.2)	68 (41.7)	0.1395
**Prevention related variables**			
History of prevention by any methods	170 (37.0)	80 (49.4)	0.0055
History of surgical mask use	176 (38.3)	88 (54.3)	0.0004
History of N95 use	7 (33.3)	8 (32.0)	0.9235
History of hepa use	1 (16.7)	3 (33.3)	0.6044
Frequency of surgical mask use at all time	66 (39.1)	37 (45.7)	0.3192

Note. Data present in number (% by no conversion or conversion group), unless indicated. Data for no conversion and conversion group may not total 461 and 163, because of missing data. History of tuberculosis in the family indicating history of tuberculosis in anyone of family members who lived in the same household, years of tuberculosis in family indicating numbers of years of history of tuberculosis in family members, and history of tuberculosis exposure in the past year indicating history of tuberculosis exposure in subjects' workplaces in the past year.

**Figure 2:**
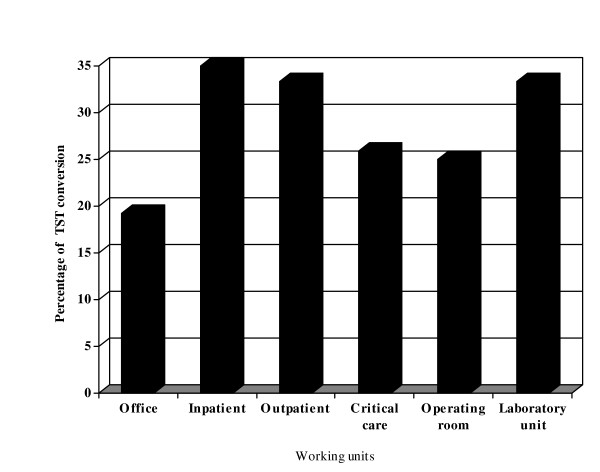
**Percentage of subjects with tuberculin skin test (TST) conversion by working units**.

Almost half of the 291 subjects or 48.7%, worked in the office unit, while 143 (22.9%), 84 (13.5%), 27 (4.3%), 52 (8.3%), and 27 (8.3%) worked at inpatient, outpatient, intensive care, operating room, and laboratory units. Of the subjects who worked in the inpatient, outpatient, intensive care, and operating room units 34.6%, were nurses, 34.6% were nurse assistants and 30.8% were ward staff, while subjects from the laboratory units were all lab technicians. There were three working units that had TST conversion rates of more than 30% including the inpatient, outpatient, and laboratory units (Figure 2).

Univariate analyses showed that factors significantly associated with having TST conversion were the working unit, history of tuberculosis exposure in the past year, and history of prevention by any method or by surgical masks (Table 2). There were only two factors that remained in the final model predictive of having TST conversion; the history of tuberculosis exposure in the past year and the working unit (Table 2). Only inpatient and outpatient units, however, were statistically significant with an adjusted odds ratios [95% confidence interval] of 1.99 [1.25–3.17] and 1.91 [1.10–3.17]. For the final model, the Hosmer-Lemeshow values and the c values were 0.03 (p value 0.99) and 63.3.

**Table 2:** Results of univariate and multivariate regression analyses showed independent variables and their crude odds ratios (ORs) and adjusted odds ration (Adjusted ORs) with 95% confidence interval (95% CI) for having a tuberculin test conversion.

Variables	ORs (95%CI)	Adjusted ORs (95%CI)
Working unit		
Office	1.00	1.00
Inpatient unit	2.26 (1.44–3.54)	1.99 (1.25–3.17)
Outpatient unit	2.10 (1.22–3.60)	1.91 (1.10–3.17)
Critical care unit	1.69 (0.59–3.64)	1.31 (0.52–3.30)
Operating room	1.40 (0.70–2.80)	1.50 (0.77–3.12)
Laboratory unit	2.10 (0.90–4.92)	2.04 (0.84–4.96)
History tuberculosis exposure in the past year	2.46 (1.63–3.72)	2.26 (1.47–3.47)
History of prevention by any method^a^	1.66 (1.16–2.39)	Not retained
History of surgical mask use^a^	1.92 (1.34–2.76)	Not retained

**Note**. ^a^not retained in the final model by multivariate logistic regression analyses

## Discussion

The prevalence of TST conversion in HCW was about one-fourth of the total tested subjects. A history of tuberculosis exposure in the past year and working in either the inpatient or outpatient units were predictive for being TST conversion. This is the first study that indicated that workplaces were significantly associated with TST conversion in an endemic area of tuberculosis. These particular working areas, inpatient or outpatient units, should be monitored closely in tuberculosis control programs of health care facilities.

Srinagarind Hospital, a university hospital, is a tertiary care and teaching hospital with 800 beds, located in the Northeastern part of Thailand. The World Health Organization (WHO) report indicates an incidence rate of new and relapsed tuberculosis in 2006 was 89/100,000 population [12]. Similar to other ASEAN countries, Thailand is included in the list of twenty-two tuberculosis high-burden countries by the WHO.

The results of this study indicated that working in health care facilities, particularly the in- and out-patient units, is significantly associated with recent onset LTB. Working in either place increased the risk of TST conversion of 99% and 91%, by the adjusted odds ratio (Table 2). These risks are statistically significant for the working unit in multivariate analysis. Both workplaces have been ignored regarding tuberculosis prevention programs in most health care facilities. Not surprisingly, the critical care unit and operating rooms were not associated with new tuberculosis infection. Both places had negative air pressure systems which could prevent spreading of tuberculosis. A previous report from Brazil [13] did not find the workplace a significant factor in TST conversion.

A history of tuberculosis exposure in the workplace increased the risk of TST conversion about 2.3 times. Even though a history of prevention, particularly using surgical masks in TST conversion subjects, as shown by univariate analysis (Table 2), was, unfortunately, not an effective prevention. Previous reports showed that wearing surgical masks did not prevent tuberculosis infection. Masks were designed to stop droplet nuclei from being spread into the air by person wearing them [14]. In the University hospital, HCWs were wearing surgical or other masks only when they were caring for a suspected tuberculosis patient.

## Conclusion

This study showed that some workplaces in health care facilities in Thailand do increase risks of latent tuberculosis in health care workers, particularly in the in- and out-patient units. Policy development regarding tuberculosis infection control programs focused on workplace prevention in health care facilities in Thailand is needed.

## Competing interests

The authors declare that they have no competing interests.

## Authors' contributions

KS designed study, collected and validated data, performed statistical analysis, and drafted the manuscript. NC and KT participated in its design and coordination and helped to draft the manuscript. KaS and PL performed statistical analysis and helped to draft the manuscript. JB carried out the TST and collected data. WR validated data. All authors read and approved the final manuscript.

## References

[B1] Detection and treatment of latent tuberculosis infection in Massachusetts college and university students. Medical Advisory Committee for the Elimination of Tuberculosis (MACET). http://www.mass.gov/Eeohhs2/docs/dph/cdc/tb/college_students_guide.pdf.

[B2] Comstock GW, Livesay VT, Woolpert SF (1974). The prognosis of a positive tuberculin reaction in childhood and adolescence. Am J Epidemol.

[B3] Sutherland I (1976). Recent studies in the epidemiology of tuberculosis, based on the risk of being infected with tubercle bacilli. Adv Tuberc Res.

[B4] U.S. Department of health and human service, Centers for Disease Control and Prevention. Self-study modules on tuberculosis: module 3. Targeted testing and the diagnosis of latent tuberculosis infection and tuberculosis disease. Atlanta, Georgia, 2008. http://www.cdc.gov/TB/education/ssmodules/pdfs/Module3.pdf.

[B5] Mirtskhulava V, Kempker R, Shields KL, Leonard MK, Tsertsvadze T, del Rio C, Salakaia A, Blumberg HM (2008). Prevalence and risk factors for latent tuberculosis infection among health care workers in Georgia. Int J Tuberc Lung Dis.

[B6] Vinton P, Mihrshahi S, Johnson P, Jenkin GA, Jolley D, Biggs BA (2009). Comparison of QuantiFERON-TB Gold In-Tube Test and Tuberculin Skin Test for Identification of Latent Mycobacterium tuberculosis Infection in Healthcare Staff and Association Between Positive Test Results and Known Risk Factors for Infection. Infect Control Hosp Epidemiol.

[B7] Kayanja HK, Debanne S, King C, Whalen CC (2005). Tuberculosis infection among health care workers in Kampala, Uganda. IntJ Tuberc Lung Dis.

[B8] Swinker M (1997). Occupational infections in health care workers: prevention and intervention. Am Fam Physician.

[B9] Joshi R, Reingold AL, Menzies D, Pai M (2006). Tuberculosis among health-care workers in low- and middle-income countries: a systematic review. Plos Med.

[B10] Hosmer DW, Hosmer T, Le Cessie S, Lemeshow S (1997). A comparison of goodness-of-fit tests for the logistic regression model. Stat Med.

[B11] Hanley JA, McNeil BJ (1982). The meaning and use of the area under a receiver operating characteristic (ROC) curve. Radiology.

[B12] World Health Organization (WHO) Global tuberculosis control – surveillance, planning, financing WHO Report 2006. http://www.who.int/globalatlas/dataQuery/default.asp.

[B13] Roth VR, Garrett Do, Laserson KF, Starling CE, Kritski AL, Medeiros EA, Binkin N, Jarvis WR (2005). A multicenter evaluation of tuberculin skin test positivity and conversion among health care workers in Brazilian hospitals. Int J Tuberc Lung Dis.

[B14] U.S. Department of health and human service, Centers for Disease Control and Prevention. Self-study modules on tuberculosis: module 5. Infectiousness and infection control. Atlanta, Georgia, 2008. http://www.cdc.gov/TB/education/ssmodules/pdfs/Module5.pdf.

